# On the Role of Institutional Logics in Legitimacy Evaluations: The Effects of Pricing and CSR Signals on Organizational Legitimacy

**DOI:** 10.1177/01492063211070274

**Published:** 2022-03-22

**Authors:** Alex Bitektine, Fei Song

**Affiliations:** 5618 Concordia University; Ryerson University

**Keywords:** institutional theory (sociology), sustainability, cognition/cognitive processes, corporate social responsibility, attitudes

## Abstract

The relationship between institutional logics and organizational legitimacy remains largely unaddressed in organizational theory and management research. We explore how individual evaluators primed with a particular institutional logic react to organizational signals sent by a firm's product/service pricing and by its engagement in corporate social responsibility (CSR) activities. In three experimental studies, we identify how the activation of a market logic or a family logic in evaluators’ minds moderates the effect of pricing and CSR engagement signals on their judgments of legitimacy of a firm, as well as on their behavioral intentions. An unexpected finding from our study was that, while participants primed with the family logic reacted positively to a CSR engagement signal sent by the firm but remained indifferent to a market-based premium-pricing signal, those primed with the market logic reacted positively to both premium-pricing and CSR engagement signals, suggesting that CSR engagement forms part of their understanding of the market logic.

## Introduction

Institutional logics, which encompass assumptions, values, beliefs, and rules that guide social actors’ behaviors and provide meaning to their social reality (Thornton & Ocasio, 1999), have been identified as fundamentally important latent variables (Thornton, Ocasio, & Lounsbury, 2012), affecting individuals’ goals, attitudes, and actions (Aarts & Dijksterhuis, 2003; Glaser, Fast, Harmon, & Green, 2017; Lounsbury, 2007). Since different institutional logics activate different sets of values, goals, and schemas (Thornton et al., 2012) in an individual's mind and are theorized to affect attitudes (Lounsbury, 2007; Thornton et al., 2012), it can be expected that they will have a profound impact on organizational legitimacy judgments and thus determine whether the organization is seen as acceptable and appropriate within the system of norms, values, and beliefs of evaluators (Suchman, 1995). However, the interaction of institutional logics and legitimacy—one of the foundational concepts of institutional theory—has yet to receive its due attention in the extant research. The inattention to this interaction in the extant literature is particularly surprising, given that legitimacy evaluations are essential for organizations’ performance and survival (Baum & Oliver, 1991; Rao, 1994) and institutional logics are theorized to play an important role in defining individuals’ goals, attitudes, and actions (Aarts & Dijksterhuis, 2003; Glaser et al., 2017; Lounsbury, 2007; Thornton et al., 2012).

At the same time, an extensive body of macro-social research theoretically differentiates various kinds of institutional logics (Besharov & Smith, 2014; Ocasio, Lowenstein, & Nigam, 2015; Thornton et al., 2012) and describes one logic replacing another (Thornton & Ocasio, 1999), logics being combined to create new “hybrid” logics (Busco, Giovannoni, & Riccaboni, 2017; Jay, 2013), or modified to include new elements (Busco et al., 2017; Haveman & Rao, 2006). However, in the absence of empirically grounded understanding of which socio-cognitive elements, such as identities, values, goals, and schemas (Thornton et al., 2012), belong to which logic, there is no empirically verifiable way to identify which of the three situations described above researchers are facing. Thus, understanding what elements a particular institutional logic includes for social actors (or, in other words, how they understand it) represents another important yet largely overlooked question in empirical research on institutional logics.

To address this research agenda, we explore how the activation of a particular institutional logic in individual evaluators’ minds affects their legitimacy judgments made in reaction to common organizational signals sent by a firm's product/service pricing and by its engagement in corporate social responsibility (CSR) activities. Such an exploration allows us not only to shed light on how institutional logics contribute to the diversity in evaluators’ judgments of firms’ legitimacy (Bitektine & Haack, 2015) but, more importantly, also to gain insight into institutional logics as complex sociocognitive structures and explore their constitutive elements.

In three experimental studies, we demonstrate how the activation of a market logic or a family logic in evaluators’ minds moderates the effect of pricing and CSR signals on their legitimacy judgments of a firm, as well as how it affects their choice of a firm in the context of competitive bidding for a major construction project. We find an inverted U-shape relationship between a firm's pricing and legitimacy judgments, which suggests that only moderate price premiums signal legitimacy. Our findings also suggest that CSR engagement forms part of our study participants’ understanding of the market logic. An important managerial and educational implication for this finding is that the goals of CSR and sustainable development are likely to be better met not by attempting to invoke some socially oriented institutional logic (e.g., family, community, social welfare, etc.) in business settings, where contextual cues for the activation of such logics are scarce, but rather by redefining the understanding of the market logic (the most prevalent logic in business settings) in such a way that CSR and sustainability considerations form an integral part of it. While the CSR research currently experiences disenchantment with building the “business case for CSR,” “win-win theories,” or “eco-efficiency” arguments (Barnett, 2019; King & Pucker, 2021; Margolis & Walsh, 2003), our findings suggest that the association of the cognitive schema of CSR engagement with the market logic does exist in the minds of our participants and affects their judgments and behavioral intentions, regardless of whether the firm can profit by responding to the societal concerns.

Since “regularities underlying organizational dynamics change over time such that empirical generalizations that are true during one period may be false in a different period” (Davis, 2010: 690), the exploration of socio-cognitive structures, such as institutional logics and social judgments, is bound to be affected by change of generations. The effect of generations is even more important, given that institutional logics are known to change over time both in terms of content (Ioannou & Serafeim, 2015) and in terms of their relative importance in society (Dunn & Jones, 2010). For this reason, to ensure managerial relevance and predictive power of our study, we chose to focus on new entrants into the labour force as our research participants. We believe such a focus in this study is warranted, as it allows us to see how the new generation of employees and managers reacts to specific social signals sent by firms and how it understands different institutional logics. The results we report here are thus generalizable to the new generation of employees and managers.

## Background and Theory Development

### Institutional Logics

Institutional logics, or “socially constructed, historical patterns of material practices, assumptions, values, beliefs, and rules by which individuals produce and reproduce their material subsistence, organize time and space, and provide meaning to their social reality” (Thornton & Ocasio, 1999: 804), play an important role in defining individuals’ attitudes and actions (Aarts & Dijksterhuis, 2003; Glaser et al., 2017; Lounsbury, 2007; Thornton et al., 2012). They constrain the means and ends of social actors (Besharov & Smith, 2014; Pache & Santos, 2013) and, at the same time, enable and constitute individual actors, organizations, and society (Thornton & Ocasio, 2008). Furthermore, they also “shape and create the rules of the game, the means-ends relationships by which power and status are gained, maintained, and lost in organizations” (Thornton & Ocasio, 2008: 112). As a result, institutional logics fundamentally influence stability and change in organizations and society. The presence of certain institutional logic is usually inferred from the analysis of discourse (Kroezen & Heugens, 2019; Thornton & Ocasio, 1999) or rich qualitative data on organizational processes (Lok, 2010; Lounsbury, 2002) and is theorized as a factor in explaining social actors’ behaviors and/or the outcomes of institutional change (Pahnke, Katila, & Eisenhardt, 2015; Vasudeva, Alexander, & Jones, 2015).

The burgeoning literature on institutional logics has described and contrasted multiple types of logics (Besharov & Smith, 2014; Friedland & Alford, 1991; Lounsbury, 2002; Pache & Santos, 2013; Thornton et al., 2012), which are formed around “a cornerstone institution that represent the cultural symbols and material practices that govern commonly recognized area of life” (Thornton et al., 2012: 54). At the macro-social level, the diversity of institutional logics forms an interinstitutional system (Thornton et al., 2012), where each institutional logic constitutes a central part of its respective “institutional order,” such as the market, the state, the family, or religion (cf. Friedland & Alford, 1991). Institutional logics research at the organization and field levels has explored the competition between conflicting institutional logics and described how these conflicts are resolved (Besharov & Smith, 2014; Friedland & Alford, 1991; Jones, Maoret, Massa, & Svejenova, 2012; Lok, 2010; Lounsbury, 2002; Pache & Santos, 2013; Thornton & Ocasio, 1999).

At the micro-social level, institutional logics are theorized to provide individual actors with ideational elements (Friedland & Alford, 1991; Kroezen & Heugens, 2019; Thornton & Ocasio, 2008), such as implicit schemas and norms, that serve as cognitive and normative tools that help actors “to make sense of their environment and to identify, express, and justify particular means and ends” (Kroezen & Heugens, 2019: 979). The scarce empirical evidence from experimental studies on the microfoundations of institutional logics suggests that the activation of particular institutions (such as norms, stereotypes, implicit theories, schemas, etc.) in the actor's mind through priming brings to the fore a complex pattern of interrelated concepts, norms, role schemas, and implicit theories (Bargh, 2006; Fiske & Taylor, 2007; Glaser et al., 2017; Thornton et al., 2012). It has an important impact on individuals’ judgments (Benard, Sierra, & Ysa, 2019), behaviors (Aarts & Dijksterhuis, 2003), as well as on discursive justifications that individuals invoke to explain their actions (Glaser et al., 2017).

Furthermore, logics are embedded “in the common identity of industry players” (Thornton & Ocasio, 1999: 803), and different role identities are associated with different institutional logics (Creed, DeJordy, & Lok, 2010; Lok, 2010). Since an individual can enact different role identities in different situations (e.g., an employee, a parent, an activist, etc.), he/she can be a carrier of a number of different institutional logics that are stored in his/her memory and are available for potential retrieval and use in specific contexts. However, “information can be stored in memory, that is be ‘available,’ but not be easily retrieved or ‘accessible’” (Higgins, 1996: 134). According to Higgins (1996), the likelihood that some stored knowledge will be activated is determined by two factors: the accessibility of the knowledge and the fit between this knowledge and the presented stimulus. Accessibility represents the “activation potential of available knowledge” (Higgins, 1996: 134), but for this potential to be realized, and thus for the knowledge structure to be activated, there should also be a fit “between the features of some stored knowledge and the attended features of a stimulus” (p. 135), and the greater this fit, the greater is the likelihood that the knowledge structure will be activated by the stimulus. Activation, in turn, further increases the accessibility of all the knowledge associated with the focal knowledge structure, which explains the mechanism of “sequential priming” (Bargh, 2006).

Since humans are usually exposed to multiple conflicting stimuli that could potentially have a priming effect, these two factors—accessibility and fit—act as filters that “reduce what ‘gets through’ to influence us” (Bargh, 2006: 158). Bargh (2006: 158) describes this element of nonconscious social behavior as “selective attention” and sees it as “a powerful tool in the reduction of the often overwhelming abundance of information available in the current environment.”

The possibility of “nonconscious activation of social knowledge structures” (Bargh, 2006: 147), which is the underlying mechanism of cognitive priming, opens up a possibility to explore the effects of institutional logics on judgments and behaviors of individuals (Benard et al., 2019). It can be expected that the fit between a stimulus and social knowledge structures highlighted by Higgins (1996) and Bargh (2006) plays an important role not only in activation of a particular institutional logic but also in interpretation of subsequent stimuli. Thus, in line with Bargh’s (2006) conception of nonconscious social behavior, it can be expected that an activation of a particular institutional logic makes individuals more receptive to those subsequent stimuli that are more consistent with this logic.

However, institutional logics are complex sociocognitive structures (Kroezen & Heugens, 2019; Lounsbury, Steele, Wang, & Toubiana, 2021; Thornton et al., 2012), and the question of which stimuli are more consistent with a particular logic cannot be meaningfully addressed without determining which elements or cognitive schemas (Glaser et al., 2017) this logic actually includes. To date, the content of institutional logics has received little empirical attention, and most of the literature relies on theoretical assumptions that a given socio-cognitive element is part of a particular logic. While many important insights came from the distinction of theorized ideal types of institutional logics (Friedland & Alford, 1991; Thornton & Ocasio, 2008; Thornton et al., 2012), further advances in research comparing and contrasting different institutional logics require an empirically grounded understanding of which socio-cognitive elements a particular logic includes and which it does not. This empirical research agenda is particularly important, given that institutional logics are not static, they are theorized to constantly evolve, interact with other logics, and form new, hybrid logic types (Busco et al., 2017; Haveman & Rao, 2006; Ioannou & Serafeim, 2015; Jay, 2013).

Recent developments in institutional logic priming (Benard et al., 2019; Glaser et al., 2017) allow us to identify elements of a given logic empirically by observing how individuals primed with different institutional logics react to specific stimuli that fit (or don't fit) with particular cognitive schemas theorized to be present within the institutional logic. Thornton and Ocasio (2008: 114) observed that institutional logics structure attention “by generating a set of values that order the legitimacy, importance, and relevance of issues and solutions.” Therefore, we can expect that social signals addressing cognitive schemas that are absent in a given institutional logic will be ignored as irrelevant, whereas signals addressing schemas that form part of the activated logic will be perceived as important and relevant and thus will affect the judgments and behavior of individuals primed with it. In other words, by priming people with a particular institutional logic and then exposing them to different social stimuli that are theorized to be related to one or the other institutional logic (e.g., pricing or CSR signals—see below), we can empirically verify which signals a particular institutional logic is sensitive to and thus identify which cognitive schemas it includes.

### Organizational Legitimacy

Since institutional logics play an important role in defining individuals’ goals, attitudes, and actions (Aarts & Dijksterhuis, 2003; Glaser et al., 2017; Lounsbury, 2007; Thornton et al., 2012), it is conceivable that institutional logics will have an important effect on organizational legitimacy—one of the foundational social evaluation concepts in institutional theory (Meyer & Rowan, 1977; Suchman, 1995; Suddaby, Bitektine, & Haack, 2017). However, the interaction of institutional logics and legitimacy has yet to receive its due attention in the extant research.

Following Suchman (1995: 574), legitimacy can be defined as “a generalized perception or assumption that the actions of an entity are desirable, proper, or appropriate within some socially constructed system of norms, values, beliefs, and definitions.” While the literature discerns multiple subtypes of organizational legitimacy (Aldrich & Fiol, 1994; Bitektine, 2011; Ruef & Scott, 1998; Suchman, 1995), we are particularly interested here in assessment of the organization as normatively appropriate and conforming to social norms, recognized principles, or accepted standards, which has been associated with the most common “sociopolitical” (Aldrich & Fiol, 1994) or “moral” (Suchman, 1995) legitimacy subtype. Following Aldrich and Fiol (1994), hereafter we refer to this subtype as “sociopolitical legitimacy” and note that the findings of this study should not be generalized to Suchman’s (1995) cognitive and pragmatic legitimacy subtypes without further empirical research, as these subtypes of legitimacy are associated, respectively, with cognitive taken-for-grantedness or with evaluator's self-interest calculations and are thus fundamentally different from sociopolitical legitimacy judgments.

The growing literature on legitimacy (of different types) has drawn attention to how social actors—individuals, groups, organizations, and society at large—make judgments about organizations, as well as how organizations, in turn, seek to influence this process and how they benefit from positive assessments by their audiences (Bitektine, 2011; Suchman, 1995; Suddaby & Greenwood, 2005). From the perspective of organizations being evaluated, legitimacy is viewed as a critical organizational resource enhancing performance and survival (Ashforth & Gibbs, 1990; Baum & Oliver, 1991; Dowling & Pfeffer, 1975; Pollock & Rindova, 2003; Singh, Tucker, & House, 1986). As a result, organizations are interested in preserving and/or increasing their legitimacy, and they often put a special effort in sending positive signals to the evaluators to influence their judgments (Connelly, Certo, Ireland, & Reutzel, 2011; Spence, 1974). While there is some evidence that such signals can be effective at improving legitimacy of an organization (Certo, 2003; Higgins, Stephan, & Thursby, 2011; Kim & Jensen, 2014), little is known about how different signals sent by business organizations affect evaluators’ legitimacy judgments and what role the activation of a particular institutional logic in the minds of evaluators plays in this process.

**Figure 1:**
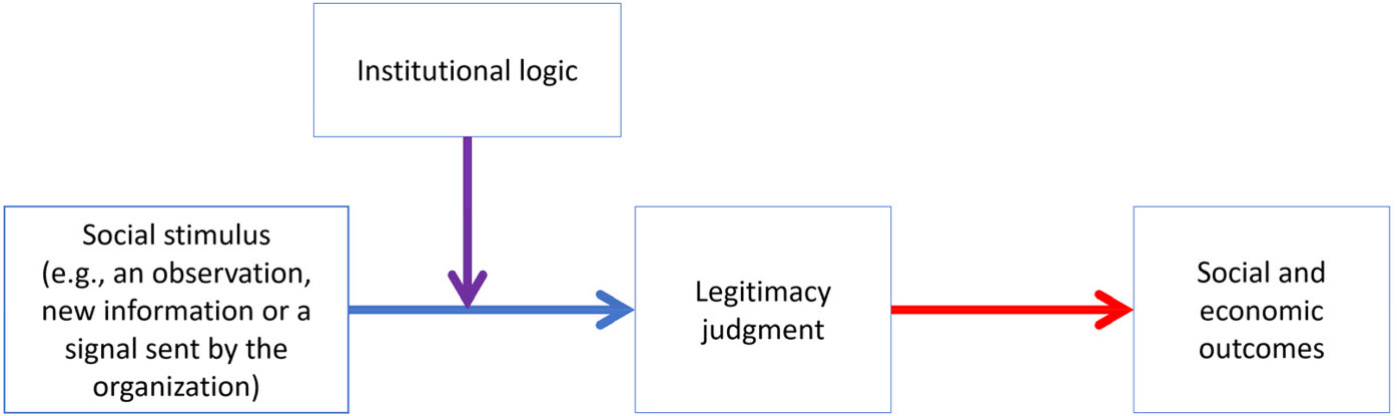
Theorized Relationship Between Institutional Logic and Legitimacy

The interaction of institutional logics with legitimacy evaluations is theoretically plausible, since institutional logics are theorized to play an important role in defining individuals’ attitudes and actions (Aarts & Dijksterhuis, 2003; Glaser et al., 2017; Lounsbury, 2007; Thornton et al., 2012). By activating and thus making more available certain norms, values, and cognitive schemas, institutional logics essentially select which set of norms, values, beliefs, and definitions is more likely to be used by evaluators in making their sociopolitical legitimacy judgments (cf. Bitektine & Haack, 2015), as well as which features and actions of the organization are perceived as relevant and important for such an evaluation. This suggests that at the micro-social level, institutional logics influence legitimacy judgments that evaluators make in response to some social stimuli, such as observations of the organization's properties, behaviors, or outcomes. Figure 1 summarizes the proposed relationship between social stimuli coming from the external environment, institutional logics, and legitimacy judgments of individual evaluators.

The exploration of the relationships outlined in Figure 1 in experimental settings requires (1) priming evaluators with one or another institutional logic, (2) exposing them to different social stimuli that are theorized to be relevant to a particular institutional logic, and (3) measuring their sociopolitical legitimacy judgments and/or behavioral outcomes of these judgments. Thus, in this study, we set out to identify how evaluators’ sociopolitical legitimacy judgments are influenced by two fairly different kinds of stimuli that serve as common signals of a firm's social qualities—premium pricing and CSR engagement—and most importantly, to explore how the relationship between these signals and sociopolitical legitimacy judgments is affected by two different institutional logics—the market logic and the family logic (Glaser et al., 2017; Thornton et al., 2012). Below we describe in detail these constructs, as well as the theorized relationships between them, and develop hypotheses that we then test in three experimental studies.

### Firms’ Signals and Legitimacy Judgments

The two signals selected for this study—pricing and CSR engagement signals—are among the most common social signals that firms send to their audiences in competitive markets. Furthermore, these signals are theorized to differ in their relationship to institutional logics, which makes them particularly suitable for the purposes of our research.

#### Pricing signal

One of the principal signals in market settings is the pricing signal, or a change in the price of goods or services, which indicates that the supply or demand should be adjusted (Arrow, 1959). However, differences in pricing levels for the same product/service occur even in equilibrium markets. Such differences are of particular interest to sociologists and communication theorists, as they serve as indications of unobservable underlying quality or some other intrinsic value, and thus require a more nuanced interpretation by evaluators. It has been observed that firms use product price for “social signaling” (Saad, 2007: 86), which attenuates the effect of price–quality relationship. Thus, marketing literature has described the Veblen effect, where a price premium is used to signal high social status (Chao & Schor, 1998; Saad, 2007; Veblen, 1998[1899]). Furthermore, the common law of business balance, which is usually expressed as “you get what you pay for,” suggests that a low price may be interpreted as a sign that the producer may have compromised on quality, and thus negatively affect the producer's reputation (Saad, 2007).

Although prior literature has identified a positive relationship of premium pricing with status (Benjamin & Podolny, 1999; Podolny, 2005) and reputation (Boyd, Bergh, & Ketchen, 2010; Lange, Lee, & Dai, 2011), we argue that the relationship between pricing and legitimacy is more complex. First, legitimacy judgment implies comparison of the organization's performance to some conception of a norm (Bitektine, 2011; Suchman, 1995), as opposed to a direct comparison with other organizations in reputation judgments (Lange et al., 2011). While most research on legitimacy deals with situations where a social norm is part of a general cultural background and is known to the evaluator through education or socialization, in industry-specific norms, one can frequently observe a situation where an outside evaluator may not have a clear idea of what is really “normal”—for example, a lay person may have difficulty assessing what level of pollution is deemed acceptable for a steel plant or what liquidity ratio is normal for a chartered bank. In such instances, evaluators first heuristically derive some conception of a norm from observation of other actors in a set and then assess how much the focal actor deviates from such a norm (Deephouse, 1999; Miller & Chen, 1995). What is important here is that the evaluator does not directly compare the focal actor to other actor(s), as is the case in reputational judgments (Fombrun, 1996: 72) but performs a comparison of the focal entity to some conception of a norm derived from observing the average, the most commonly, or the most recently occurring value among category members (cf. the discussion of anchoring and availability heuristics in Kahneman, 2011; Tversky & Kahneman, 1974).

Second, in sociopolitical legitimacy judgments, the interpretation of the observed deviance from the norm, such as a high price, depends on whether the high price is viewed as normatively justifiable (and hence potentially signal some positive social qualities) or as excessive (and hence illegitimate—cf. common accusations of “corporate greed,” “evil profit,” or “price gouging”). The difference between these two extremes in sociopolitical legitimacy judgments implies the activation of different cognitive mechanisms in interpreting the pricing signal: either the development of a “normalizing” account explaining and justifying the high price, or a “moral outrage” (Hafenbrädl & Waeger, 2017) attributing the price difference to bad corporate behavior. The system justification theory (Jost, 2001; Jost, Banaji, & Nosek, 2004) would suggest that “people have a desire to defend and legitimize the current state of affairs” (Proudfoot & Kay, 2014: 176) and would use “information about how things are currently done to inform their beliefs about how things should be done” (Proudfoot & Kay, 2014: 176). To interpret the price premium, evaluators are more likely to search for some superior intrinsic value or other reason that justifies the firm's higher price; that is, they will interpret the higher price as a signal of some underlying superior qualities of the firm. If such a search for superior qualities fails to generate a plausible explanation, the high price is likely to be perceived as excessive and be interpreted as an apparent violation of some moral standard or principle (Hafenbrädl & Waeger, 2017), as a manifestation of counternormative behavior. Therefore, we hypothesize that there is an inverted U-shape relationship between pricing and sociopolitical legitimacy, and hence, only moderate premium pricing will be positively related to sociopolitical legitimacy judgment, while extreme pricing level will be perceived as illegitimate. Thus,

*H1a*: Moderate premium pricing will be positively related to sociopolitical legitimacy judgment.
*H1b*: Extreme premium pricing will be negatively related to sociopolitical legitimacy judgment.

#### CSR engagement

The other signal that presents a particular theoretical interest is firms’ engagement in CSR activities. CSR “consists of clearly articulated and communicated policies and practices of corporations that reflect business responsibility for some of the wider societal good” (Matten & Moon, 2008: 405). The concept of CSR reflects the belief that the responsibility of firms for their actions and practices extends beyond the profitability concerns of shareholders to diverse issues affecting employees, their families, communities, and a broader society (Carroll, 1999; McWilliams & Siegel, 2001). CSR engagement has been shown to be a powerful signal associated with greater legitimacy and social approval (Garriga & Melé, 2004; Hart, 1995; Joutsenvirta & Vaara, 2015; Matten & Moon, 2008). Overall, it has been argued that CSR provides protection to a firm's relationship-based intangible assets (Godfrey, 2005), justifies higher prices (Koschate-Fischer, Huber, & Hoyer, 2016), increases firm performance (Hillman & Keim, 2001), and may even create a competitive advantage (McWilliams & Siegel, 2001; Porter & Kramer, 2006).

It should be noted, however, that the interpretation of CSR as a signal has been changing over time. Ioannou and Serafeim (2015) observed that market analysts’ reaction to firms’ CSR ratings has evolved substantially since 1990s, when CSR was interpreted negatively, and meeting the expectations of nonshareholding stakeholders was viewed as destroying shareholder wealth. The authors suggest that since then CSR engagement has been reinterpreted “as a legitimate part of corporate strategy, minimizing operational risks and even contributing positively towards long-term financial performance” (Ioannou & Serafeim 2015: 1058). Recent studies also observe that CSR as a signal has become an important part of corporate communications (Haack, Schoeneborn, & Wickert, 2012; Schultz & Wehmeier, 2010) and that managers now have an established belief in the business case for CSR (Hafenbrädl & Waeger, 2017).

Given these observations, we expect a positive effect of the CSR engagement signal on firms’ sociopolitical legitimacy (Garriga & Melé, 2004; Hart, 1995; Matten & Moon, 2008):

*H2:* Perceived CSR engagement will be positively related to sociopolitical legitimacy judgment.

### Institutional Logics and Legitimacy Judgments

#### Market versus family logics

Among multiple types of institutional logics that constitute the interinstitutional system (Thornton et al., 2012), we have identified two logics that are already associated with established manipulation instruments and validated operational measures in the literature (Glaser et al., 2017): (1) *the market logic*, which is one of the best described and most studied logics with a much clearer delineated scope relative to other types of institutional logics (Benard et al., 2019; Lamont, 2012; Meyer, Egger-Peitler, Hollerer, & Hammerschmid, 2014; Qiu, Gopal, & Hann, 2017) and (2) *the family logic* (Fairclough & Micelotta, 2013; Glaser et al., 2017; Thornton et al., 2012), which is associated with a fundamentally different set of norms, values, and beliefs and thus provides a sufficient contrast with the ideational content of the market logic. We describe the two logics in greater detail below.

As Friedland and Alford (1991: 234) put it, “a market <…> is not simply an allocative mechanism but also an institutionally specific cultural system for generating and measuring value.” The market logic thus represents the central logic of the market order in society (Friedland & Alford, 1991). Under the market logic, the value is seen in profit maximization (Lamont, 2012), financial controls and efficiency (Benard et al., 2019), as well as in the pursuit of self-interest and competition (Thornton & Ocasio, 1999). The relationships between actors under this logic are constructed through the prism of market settings and a root metaphor of economic transactions (Thornton et al., 2012).

In contrast, family logic is central to another fundamental institutional order of society—the nuclear family. This logic is defined by its focus on “community and the motivation of human activity by unconditional loyalty to its members…” (Friedland & Alford, 1991: 248). It is associated with the roles of a parent, a child, a brother, a relative, as well as a number of social schemas and scripts associated with home, mutual support, and affective ties (Glaser et al., 2017). The relationships between actors under the family logic are constructed by the principles of solidarity, care, and support (Jancsary, Meyer, Höllerer, & Barberio, 2017), ideas of nurturing, conservation, and affective ties (Miller, Le Breton-Miller, & Lester, 2011), as well as loyalty of family members to each other (Glaser et al., 2017; Thornton et al., 2012). The fundamental concern for others, or “other-interest” (Glaser et al., 2017), which lies at the core of the ideational content of the family logic, comes in stark contrast with the market logic's focus on self-interest and competition and, at the same time, aligns the family logic with other types of *socially oriented logics* that emphasize empathy, cooperation, and the common good, such as community logic (Marquis & Lounsbury, 2007; Venkataraman, Vermeulen, Raaijmakers, & Mair, 2016), civic logic (Boltanski & Thévenot, 2006; Lamont, 2012), collective activity logic (You & Guzman, 2019), social gain logic (Doliwa, 2019), or corporatist logic (Vasudeva et al., 2015). For illustrative purposes, we provide in Appendix 1 descriptions of some of the types of socially oriented logics commonly found in the literature and highlight common themes in these descriptions. While the exploration of the nuanced differences between these logics is beyond the scope of our paper, we draw here on family logic as a member of this broader “constellation” (Goodrick & Reay, 2011) of socially oriented logics that share the emphasis on empathy, cooperation, the common good, and concern for others and contrast it with the self-interest focused market logic (Glaser et al., 2017; Goodrick & Reay, 2011; Marquis & Lounsbury, 2007).

#### Institutional logics and social signals

The activation of either the market or the family logics can be expected to make individual evaluators more receptive to those social signals that are more consistent with the activated logic and thus prompt them to make different legitimacy judgments and decisions with respect to the same organization. Since pricing signals occur in market settings and pricing is an essential attribute of market transactions, one can expect that pricing signals will be more consistent with the market logic and thus elicit a stronger response from evaluators primed with the market logic than from those primed with the family logic. However, in light of Hypothesis 1, we expect such a relationship to hold only for moderate price premiums, since “excessive” pricing will likely cause a “moral outrage.” Therefore, we hypothesize the following moderating effects of the market logic on evaluators’ social judgments:

*H3:* The market logic, relative to the family logic, positively moderates (strengthens) the relationship between the (moderate) premium pricing signal and sociopolitical legitimacy judgment.

At the same time, CSR signals are associated with social approval by socially oriented stakeholders, such as local communities, families, or the general public. Furthermore, the focus of the family logic on nurturing future generations (Miller et al., 2011) and the multigenerational perspective it implies (cf. old family firms) is congruent with the concern of CSR and sustainable development initiatives with well-being of both current and future generations (Kim, Bansal, & Haugh, 2019). Thus, we expect that the CSR signals will have a greater effect on individuals primed with the family logic than with the market logic. Therefore,

*H4:* The family logic, relative to the market logic, positively moderates (strengthens) the relationship between the CSR signal and sociopolitical legitimacy judgment.

Thus, we theorize that in interpretation of social signals sent by firms’ pricing or by their CSR engagement, the dominant institutional logic that is activated in the mind of an evaluator has an important moderating effect on sociopolitical legitimacy judgments about the firm. Furthermore, evaluators’ response to different types of stimuli consistent (or inconsistent) with the primed institutional logic may provide important insights on what socio-cognitive elements the logic includes and what elements are not associated with a particular logic in the evaluators’ minds.

Finally, since the extant research suggests the importance of legitimacy judgments for decisions that evaluators make with respect to the focal organization (Suchman, 1995), it is important to verify empirically whether sociopolitical legitimacy mediates the effect of firms’ signals, such as pricing or CSR engagement, on the evaluators’ decision to select a particular firm's services. Therefore, we hypothesize that:

*H5:* Sociopolitical legitimacy judgment mediates the effect of the (moderate) pricing signal on the choice of the firm.
*H6:* Sociopolitical legitimacy judgment mediates the effect of the CSR signal on the choice of the firm.

**Figure 2:**
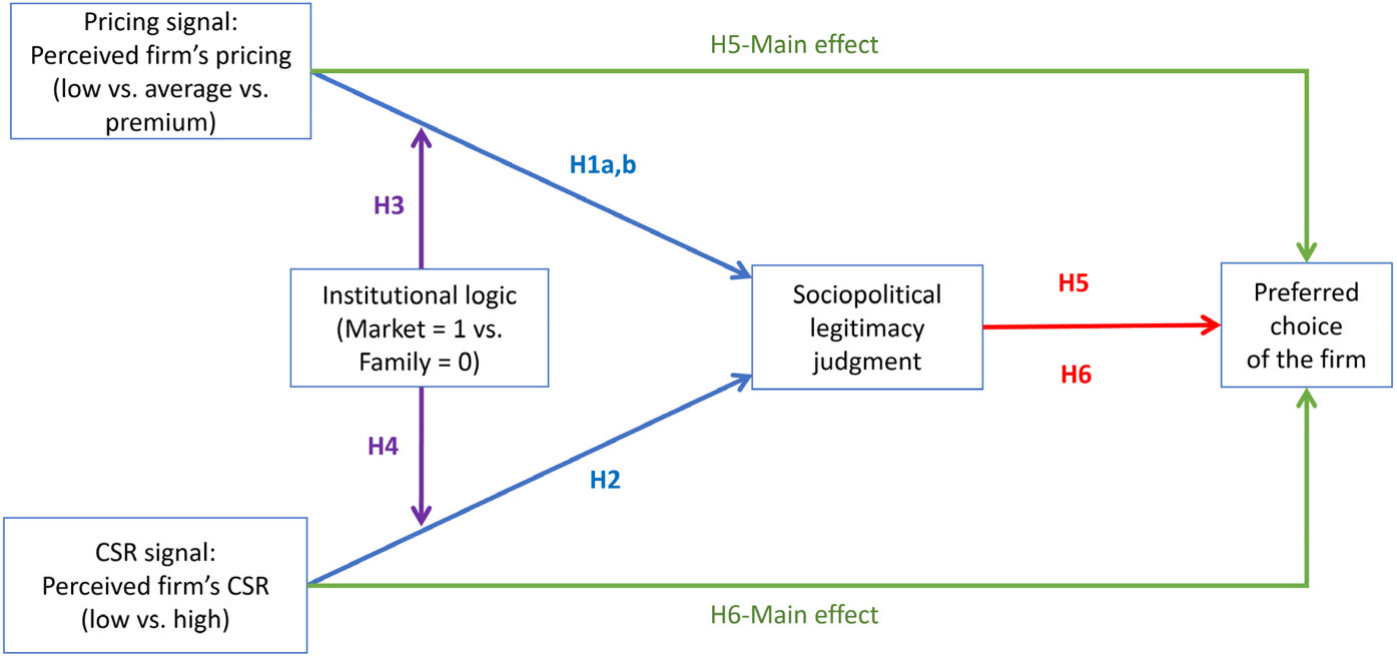
An Overview of Hypotheses

Our six hypotheses are summarized in Figure 2.

## Methods

### Overview

We designed and carried out three experimental studies to test the six hypotheses. The details of each study are summarized in Table 1. Study 1 was exploratory. It tested Hypothesis 1 and demonstrated that the pricing signal is noticed by the participants. In Studies 2 and 3, in order to activate institutional logics in a nonconscious manner, we used the priming methodology, which is categorized as either subliminal, where the subject is not aware of the prime, or supraliminal, where the subject perceives the prime but does not recognize its potential influence (Bargh, 2002; Glaser et al., 2017). We follow Glaser et al. (2017) in adopting the methodological approach of dynamic constructivism (Hong, Morris, Chiu, & Benet-Martinez, 2000) to experimentally examine institutional logics. Specifically, building on this well-established priming manipulation, Study 2 tested Hypotheses 1, 3, and 5, while Study 3 mirrors the structure of Study 2 but uses a slightly different task that allows us to test all hypotheses formulated in our theory section.

**Table 1:** Study Overview

Study No.	Hypotheses Addressed	Experimental Conditions	IV	Moderators	Mediators	DV	Key Findings
1	H1a, b	Within-person pricing manipulation: (1) Average (2) 11% above avg. (3) 57% above avg.	H1a, b: Pricing	N/A	N/A	Sociopolitical legitimacy judgment	There is a nonlinear, inverted U-shape, relationship between pricing increase and sociopolitical legitimacy judgment.
2	H1a, 3, and 5	Within-person pricing manipulation: (1) 12% below avg. (2) Average (3) 11% above avg. Between-person priming of the institutional logic: (1) Market logic (2) Family logic	H1a: Pricing H3: Pricing H5: Pricing	N/A Institutional logic N/A	N/A N/A Sociopolitical legitimacy judgment	Sociopolitical legitimacy judgment Sociopolitical legitimacy judgment Preferred choice of the firm	Moderate premium pricing is positively related to the sociopolitical legitimacy judgment. The market logic positively moderates (strengthens) the relationship between the (moderate) pricing signal and sociopolitical legitimacy judgment.
3	H1a, 2, 3, 4, 5, and 6	Within-person pricing manipulation: (1) 12% below avg., (2) Average, (3) 11% above avg. Within-person CSR manipulation: (1) High CSR rating, (2) Low CSR rating. Between-person priming of the institutional logic: (3) Market logic (4) Family logic	H1a/2: Pricing/CSR H3/4: Pricing/CSR H5/6: Pricing/CSR	N/A Institutional logic N/A	N/A N/A Sociopolitical legitimacy judgment	Sociopolitical legitimacy judgment Sociopolitical legitimacy judgment Preferred choice of the firm	Moderate premium pricing is positively related to the sociopolitical legitimacy judgment. Perceived CSR engagement is positively related to sociopolitical legitimacy judgment. The market logic (but not family logic) positively moderates (strengthens) the relationship between the (moderate) pricing signal and legitimacy judgment. Sociopolitical legitimacy judgment mediates the effect of the (moderate) pricing/CSR signal on the choice of the firm.

### Methodological Rationale and Considerations

Experiments are a common method for exploration of heuristics (Kahneman & Tversky, 1996; Tversky & Kahneman, 1974), as well as social judgments (Hahl & Zuckerman, 2014; Lount & Pettit, 2012; Pettit, Yong, & Spataro, 2010; Piff, Stancatoa, Côté, Mendoza-Dentona, & Keltnera, 2012; Rucker & Galinsky, 2008). Recent developments in micro-foundations of social evaluations are also increasingly relying on experimental methods as an important driver for inquiry (Schilke, 2018; see Bitektine, Lucas, & Schilke, 2018 for a review). In this paper, we used a controlled laboratory environment, enabling us to “make strong inferences about the causal chain of events” (Spencer, Zanna, & Fong, 2005: 846). Moreover, we employed work scenarios, which enhanced experimental realism and allowed us to manipulate conditions for both internal and external validity (Aguinis & Bradley, 2014). Our controlled experimental environment allowed us to distinguish the effects of social signals and institutional logics from other, potentially confounding factors in organizations, such as the resources exchanged or the substantive context of exchange, thereby minimizing the influence of nuisance factors that may be extremely difficult to isolate in the field (e.g., Camerer, 2003, 2015; Falk & Heckman, 2009; Fehr & Gintis, 2007; Murnighan & Wang, 2016).

A central methodological feature in our experimental design is priming. Prior work on experimentally manipulated priming supports the notion that priming exercises can effectively induce real-world mindsets (Brewer & Gardner, 1996; Cadsby, Servátka, & Song, 2013; Galinsky, Gruenfeld, & Magee, 2003; Lount & Pettit, 2012). Employing priming as one of our major manipulations, we provide parsimonious, accurate, and causal tests of people's judgments and behavior (Camerer, 2003; Fehr & Gintis, 2007; Murnighan & Wang, 2016).

We also incorporated both within- and between-person manipulations, which offers several advantages. First, having the same individual participant reviewing and evaluating different firms with pricing and/or CSR differentials allows us to control for idiosyncratic variability across evaluators to a greater extent than random assignment. For a given sample size, this method, coupled with the empirical panel-data analysis, results in greater statistical power. Second, the between-person design allows us to focus on the effect of differing institutional logics (manipulated via priming) on judgments and behavior.

Given the nature of the lab experimental method, one possible concern is that experiments such as ours may not entirely capture the complexity of real-world organizational settings and thus limit the generalizability of our results. However, the unique strength of the lab experimentation is not to recreate the real world but to establish causality and to uncover causational mechanisms, which are often extremely difficult, if not impossible, to detect and isolate in field studies. In the context of institutional theory research, earlier work has successfully demonstrated that lab experimentation can indeed advance our study and understanding of key aspects of institutional theory and its micro-foundations (e.g., Glaser et al., 2017; Levine et al., 2014; Lucas, 2003; Schilke, 2018; Zucker, 1977). Our current research builds on and extends this promising new area of research.

### Study One

The purpose of this exploratory study was to test Hypothesis 1, which postulates an inverted U-shape relationship between pricing and sociopolitical legitimacy judgment and empirically establish the level of price increase that evaluators would consider “moderate” in the context of our experiments—that is, not too high to cause a “moral outrage” but sufficiently noticeable to influence the evaluators’ legitimacy evaluations and decisions. We also sought to test the decision-making scenario to be used in subsequent experiments and the instrument to measure a firm's sociopolitical legitimacy.

#### Key variables, manipulations, and measurements

We employed a within-person manipulation for three levels of pricing strategy, which are at the average, 11% above, and 57% above the average. This within-person manipulation was used to control for individual idiosyncratic differences in social judgments when examining the impact of pricing differentials on such judgments. In order to isolate the pricing effect from potential ordering effects within the same individual, the ordering of the three within-person conditions was randomized and counterbalanced. However, no ordering effect was detected in our data. This allowed us to pool our data for subsequent analysis.

In this within-person, repeated-measure set-up, we presented participants with three firms at once in order to maximize the perceived differential of the pricing strategy of the three firms, while keeping all other aspects of these firms equivalent to each other. Specifically, participants were instructed with the following:

“*You are a member of a public committee in charge of selecting a contractor to build a new building for the hospital downtown Toronto. After preliminary screening and detailed analysis of the bidders, three engineering firms have been retained for further evaluation. You need to complete the evaluation process by assessing the three firms listed below based on the information provided and ranking your choices. The three selected bids meet the requirements with respect to building size, design, and functionality. On the financial side, the differences between the three bids were as follows: The bid from Firm A was approximately the average of the submitted bids; the bid from Firm B was 11% higher than the average bid price in this tender; while the bid from Firm C was 54% higher than the average bid price in this tender.”*

The order of these pricing strategies was randomized and counterbalanced.

For sociopolitical legitimacy judgment measure, we adopted a psychometrically validated (Bitektine, Hill, Song, & Vandenberghe, 2020) scale consisting of three items: *The company contributes positively to society*; *the company follows the best management practices*; and *I agree with the company's business practices*. On these three items, we asked participants to rank the three bidders as the top, the middle, or the bottom firm.

#### Participants and procedure

A total of 177 undergraduate Commerce students (69 men and 108 women with an average age of 21.35) from a large-size, public university in Canada participated in this online study in exchange for course credits. With a three-level, within-person treatment design, the number of observations was 531 (177 × 3 = 531), which was sufficient for making robust statistical inferences.

#### Results

**Figure 3:**
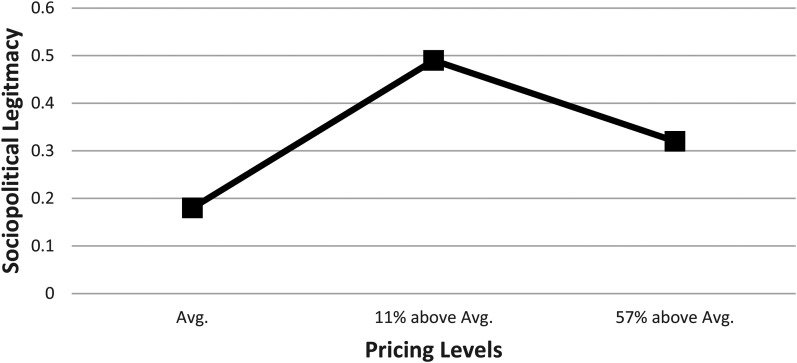
Study 1 – Proportion of Participants Ranking the Firm as Top-Ranking on Sociopolitical Legitimacy

Across the three within-person treatments, the sociopolitical legitimacy judgment measure achieved satisfactory reliability in each of the three pricing conditions (Cronbach's alpha ranges from 0.83 to 0.93). Table 2 presents descriptive statistics and correlations. Table 3 and Figure 3 provide a summary of the proportion of participants who ranked a particular firm as the top-ranking on sociopolitical legitimacy.

**Table 2:** Study 1 – Data Overview

Variable	*M*	*SD*	1.	2.	3.
1. Sociopolitical legitimacy	0.33	0.36			
2. Pricing	1.00	0.82	0.162***		
3. Gender	0.61	0.48	0.000	0.000	
4. Age	21.35	3.01	0.003	0.001	0.004

*Note*: *N* = 177. ***p < .01. Gender: female = 1 and male = 0. Pricing: average = 0, 11% above avg. = 1, 57% above avg. = 2.

**Table 3:** Study 1 – Proportion of Participants Ranking a Given Firm as the Top One (Standard Deviations in Parentheses)

	*Firm A* (Average Bid)	*Firm B* (Bid 11% Above Average)	*Firm C* (Bid 57% Above Average)	A to B Slope	B to C Slope
Sociopolitical Legitimacy	0.18 (0.27)	0.49 (0.37)	0.32 (0.34)	Positive, *p* < .001	Negative, *p* < .001

*Note*: Pricing strategies of the firms: A – Average, B – 11% above average, and C – 57% above average.

The last two columns in Table 3 present the linear slope estimations from Firm A to B and from Firm B to C. There is a nonlinear, inverted U-shape, relationship between pricing increase and sociopolitical legitimacy judgment. In particular, the linear slope from Firm A to B has a positive and significant trend, providing support to H1a, while linear slopes from Firm B to C are negative and significant, providing support to H1b.

### Study 2

The purpose of Study 2 was to empirically establish the effect of institutional logics manipulation on evaluators’ decisions. More specifically, we tested in experimental settings Hypothesis 1 (the main effect of pricing strategy on sociopolitical legitimacy judgments), Hypothesis 3 (the moderator effect of institutional logics on the aforementioned main effect), and Hypothesis 5 (the mediator effect of sociopolitical legitimacy judgments in the relationship of pricing strategy and the most favorable choice of the firm).

#### Key variables, manipulations, and measurements

We employed a combination of within- and between-person manipulation in order to manipulate the two distinct variables that we are interested in. Specifically, as in Study 1, we used the within-person manipulation of three pricing strategies adopted by a firm in its bid: low price (below average), average price, and (moderate) premium price. This within-person manipulation was used to control for individual differences in legitimacy judgments when examining the impact of pricing differentials on these judgments. In order to isolate the pricing effect from potential ordering effects within the same individual, the ordering of the three within-person conditions was randomized and counterbalanced. However, since no ordering effect was detected in our data, we pooled our data for subsequent analysis. The within-person manipulation of the pricing strategy was complemented with a between-person manipulation of institutional logic (market vs. family logic priming) to test the main effect of pricing on sociopolitical legitimacy judgments.

In this within-person, repeated-measure set-up, we presented participants with three firms at once in order to maximize the perceived differential pricing strategy of the three firms, while keeping all other aspects of these firms equivalent to each other. Specifically, participants received the same instruction as in Study 1, except that the pricing of the three firms was described as follows:

“*… The bid from Firm A was approximately the average of the two other bids; The bid from Firm B was 12% lower than the average bid price in this tender; while the bid from Firm C was 11% higher than the average bid price in this tender.”*

The order of these pricing strategies was randomized and counterbalanced.

Following Glaser et al. (2017), we asked participants in the market logic condition to write an orientation letter for new employees entering an investment banking organization, which has an overall strategic orientation of profit maximization. Specifically, participants in the market logic priming condition read the following:

“*Picture yourself as an investment banker at Goldman Sachs. Your firm operates with the primary purpose of maximizing profit. In order to do this, the CEO encourages employees to compete with each other and pursue their own self-interest. The more that employees seek to increase their own personal salaries and bonuses, the better they (and the bank) will perform. Overall, the CEO works hard to foster a culture of efficiency, self-interest, and competition. Part of your job is to embody each of these values and communicate them to incoming employees. You have been asked to write an orientation letter for several new incoming employees to let them know what it is like to work in the company. In the space below, please write out the letter that you would create in order to instill the values and expectations that the CEO has for employees.”* (adapted from Glaser et al., 2017)

For the family logic priming, we asked participants to write an orientation letter for new employees entering a family foundation organization that sought to bring honor to the family name by engaging in philanthropic work. Specifically, participants in the family logic condition read the following:

“*Picture yourself as a member of a family foundation. Your foundation is a family-based charity organization that provides financial contributions to good causes and operates with the primary purpose of bringing honor to the founding family through helping others. In order to do this, the founder encourages employees to cooperate and become friends with each other and show loyalty, both to each other and to the foundation. The more that employees seek to help others and show loyalty to the foundation, the better they (and the foundation) will perform. Overall, the founder works hard to foster a culture of care, loyalty, and treating everyone as a part of the family. Part of your job is to embody each of these values and communicate them to incoming foundation employees. You have been asked to write an orientation letter for several new incoming employees to let them know what it is like to work in the family foundation. In the space below, please write out the letter that you would create in order to instill the values and expectations that the founder has for employees.*” (adapted from Glaser et al., 2017)

In both conditions, participants were instructed to write for about 5 min and to try to provide a meaningful response during this time.

We used the same psychometrically validated scale as in Study 1 to measure sociopolitical legitimacy judgments (Bitektine et al., 2020). On these items, we asked participants to rank the three bidders as the top, the middle, or the bottom firm. Lastly, participants were also asked to select one of the three bidders as the most favorable choice for a hospital construction project. We used this item as a measure of their behavioral intention (Ajzen, 1991).

#### Participants and procedure

One hundred and seven undergraduate Commerce students (50 men and 57 women with an average age of 20.24) from a large, public university in Canada participated in this study in exchange for a $20 cash payment. With a three-level, within-person treatment design, the number of observations was 321 (107 × 3 = 321), which was sufficient for making robust statistical inferences.

Upon arriving at the laboratory, participants were seated separately and received a specific ordering of the within-person manipulations at random. After completing a consent form, we first implemented the priming manipulation. After completing their written responses to the priming instrument, participants were presented with the three firms, each associated with a different pricing strategy. Then they ranked the three firms on the three-item legitimacy judgment scale and indicated their overall choice from one of the three bidders for the project.

At the end of the session, we also collected demographic background information and administered four questions, adopted from Glaser et al. (2017) that served as manipulation checks for the institutional logic priming manipulations. Specifically, participants were asked to indicate their level of agreement on a 7-point scale (1 = *strongly disagree* to 7 = *strongly agree*) to the statement “people decide to attend university”: (1) to increase their earnings, (2) to increase their ability to contribute to society, (3) to increase their ability to succeed in a highly competitive environment, and (4) to give back to their local community.

#### Results

We first performed manipulation checks on participants’ responses to the institutional-priming manipulation. Results confirmed that the manipulations adapted from the Glaser et al. (2017) study were successful on these measures as their perceptions of the purpose of higher education were indeed significantly different across the two between-person manipulations [*M* = 5.58, *SD* = 1.43, versus *M* = 4.97, *SD* = 0.82, *t*(106) = 3.41, *p* = .008 for earning potential items, and *M* = 4.23, *SD* = 1.39, versus *M* = 5.18, *SD* = 1.08, *t*(106) = 4.68, *p* < .001 for contribution to community and society for market logic and family logic priming manipulations, respectively]. Across the between-person treatments (institutional logics) as well as across the within-person treatments (pricing), reliability of the sociopolitical legitimacy judgment achieved satisfactory reliability in each of the three pricing conditions (Cronbach's alpha ranges from 0.81 to 0.93).

Table 4 presents the data overview and Table 5 provides a summary of the proportion of participants who ranked the premium-pricing firm as the top firm on legitimacy as well as on the behavioral item of the preferred choice of the most successful bidder for the project.

**Table 4:** Study 2 – Data Overview

Variable	*M*	*SD*	1.	2.	3.	4.	5.
1. Sociopolitical legitimacy	0.56	0.49					
2. Preferred choice	0.51	0.50	0.07				
3. Pricing	1.00	0.47	−0.057	0.04			
4. Institutional logic	0.47	0.50	0.45***	0.16***	0.002		
5. Gender	0.53	0.48	−0.01	0.009	0.000	0.07	
6. Age	20.95	2.06	0.11	0.04	0.000	−0.00	−0.08

*Note*: *N* = 107. ***p < .01. Gender: female = 1 and male = 0. Pricing: below average = 0, avg. = 1, above avg. = 2. Institutional logic: market logic = 0, family logic = 1.

**Table 5:** Study 2 – Proportion of Participants Ranking the Premium-Pricing Firm as the Top One (Standard Deviations in Parentheses)

	Market-Logic Priming (*n* = 50)	Family Logic Priming (*n* = 57)	Overall (*n* = 107)
Sociopolitical legitimacy	0.61 (0.18)	0.43 (0.30)	0.51 (0.26)
Preferred choice	0.80 (0.40)	0.32 (0.47)	0.54 (0.50)

Since the key outcome variables in our study—legitimacy evaluations and behavioral ranking decisions—are both measured at the mean level, we adopted parametric analytical framework (Cohen, Cohen, Aiken, & West, 2014) for our data analysis and hypothesis tests. Specifically, we tested whether the proportion of participants who ranked the premium-pricing firm as the top firm is significantly higher than the random-chance probability of 1/3. These two types of tests yield qualitatively the same results, so we will report the parametric results below for brevity. Overall, the proportion was 51% for legitimacy judgments, and it was significantly higher than the random chance of 1/3 (*p* < .001), thus lending further support to H1a, which predicts that moderate premium pricing will be positively related to the sociopolitical legitimacy judgment.

**Figure 4:**
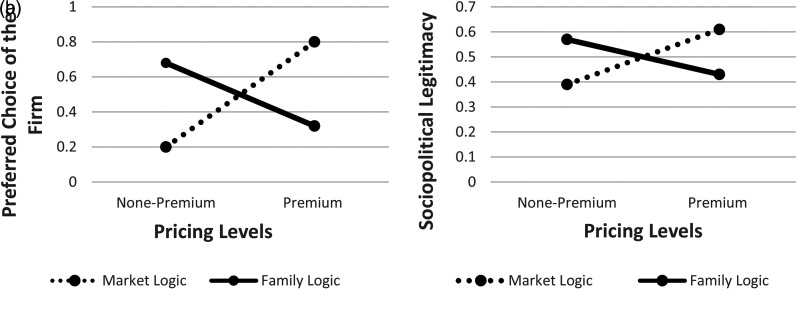
A and B: Study 2 – Proportion of Participants Ranking the Premium-Pricing Firm as the Top Choice

Next, we tested the proposed moderation effect of institutional logic priming, as outlined in H3. One-way ANOVA tests show that, compared to participants primed with the family logic, the proportion of participants primed with the market logic who ranked the premium-pricing firm as the most legitimate firm is significantly higher (0.61 vs. 0.43, *p* < .001, respectively). Thus, these results lend strong support to H3. Similarly, compared to participants primed with the family logic, significantly higher proportion of participants primed with the market logic chose the premium-pricing firm as their most favorable firm (0.80 vs. 0.32, *p* < .001). These results, visually depicted in Figure 4, reveal that institutional logics do affect individual actors’ sociopolitical legitimacy evaluations of firms.

Lastly, we explored the proposed mediating mechanisms through legitimacy on the association between pricing strategy and the choice of the firm, as outlined in Hypothesis 5. We first note that 54%, a significantly higher proportion than 1/3 (*p* < .001), of the participants selected the premium-pricing firm as their choice for the project, thus establishing the main effect for the mediation analysis. To formally test Hypothesis 5, we adopted random-effect regression models to account for the within-person, repeated-measure of panel data structure for the key behavioral measure—the choice of the firm. Specifically, we coded the variable as 1 if the firm was chosen and 0 if not. To test the indirect effect of the mediation, we adopted bootstrap analyses (Preacher, Rucker, & Hayes, 2007) to test whether the proposed mediator—sociopolitical legitimacy judgment—carries the influence of the pricing signal to the choice of the firm. However, these tests showed no indirect effect. Thus, Hypothesis 5 was not supported.

A potential explanation to the observed absence of mediation could be that price signals do not create much variance in sociopolitical legitimacy judgments as long as the price is perceived as normatively acceptable (see Study 1), and legitimacy has to do more with social approval than market differentiation (Bitektine, 2011; Suchman, 1995). To verify this conjecture, it was important to compare the effects of the market-based pricing signal with effects of another social signal that would not be associated with markets—for example, a signal coming from a more socially-oriented domain.

### Study 3

The purpose of Study 3 was thus to investigate how evaluators primed with different institutional logics respond to two different social signals—the pricing signal (as in Studies 1 and 2) and the CSR engagement signal. We theorized that the pricing signal will resonate more with evaluators under the market-logic priming (H3), while the CSR signal will show greater affinity with a more socially oriented family logic, as outlined in Hypothesis 4. Methodologically, Study 3 is an extension of Study 2, where we employed a combination of within- and between-person manipulations in order to take into account the three distinct variables that we are interested in: The pricing level and the CSR engagement are two independent variables and are manipulated in a within-person manner, and institutional logic priming is our moderator variable and is manipulated in a between-person manner. Specifically, we used the within-person manipulation for a premium-, average-, and low-pricing strategy adopted by a firm, as well as for high- and low-CSR engagement adopted by a firm. Thus, each participant was presented with six distinct firm profiles: premium pricing and high CSR, average pricing and high CSR, low pricing and high CSR, premium pricing and low CSR, average pricing and low CSR, and low pricing and low CSR. This within-person manipulation was used to control for individual differences in sociopolitical legitimacy judgments when examining the impact of pricing and CSR differentials on them. As in Studies 1 and 2, we used forced ranking to measure all items.

#### Key variables, manipulations, and measurements

All manipulations and key variables are the same as in Study 2, except the added within-person manipulation of the CSR engagement. In this within-person, repeated-measure set-up, we presented participants with two types of CSR engagement (high and low) at once, in order to maximize the perceived differential of CSR engagement among firms, while keeping all other aspects of these firms equivalent to each other. Specifically, we informed participants that a firm's CSR engagement was summarized in a numeric score, which had been compiled based on the data from firms’ CSR/sustainability reports, NGO surveillance data, and sustainability awards/rankings. Three of the six firms had high ratings ranging from 9.48 to 9.51 out of 10 on CSR performance, while the other three had low ratings ranging from 6.08 to 6.11.

#### Participants and procedure

Ninety-nine undergraduate Commerce students (40 men and 59 women with an average age of 20.59) from a large-size, public university in Canada participated in this study in exchange for a $20 cash payment. With a three-level, within-person treatment design, the number of observations was 594 (99 × 6 = 594), which was sufficient for making robust statistical inferences.

#### Results

As in Study 2, the manipulation check and reliability checks were all satisfactory. Specifically, the manipulation checks on participants’ responses to the institutional priming manipulation showed that participants’ perceptions of the purpose of higher education were significantly different across the two between-person manipulations: *M* = 6.01, *SD* = 1.35, versus *M* = 5.51, *SD* = 0.82, *t*(172) = 2.91, *p* = .004 for earning potential items, and *M* = 4.03, *SD* = 1.39, versus *M* = 5.21, *SD* = 1.18, *t*(172) = 5.98, *p* < .001 for contribution to community and society for market logic and family logic priming manipulations, respectively. Across both the between-person treatments (market vs. family logics) as well as across the within-person treatments (high vs. medium vs. low pricing and high vs. low CSR), reliability of the sociopolitical legitimacy judgment achieved satisfactory reliability (Cronbach's alpha ranges from 0.78 to 0.91).

 Table 6 provides a summary of the proportion of participants who ranked the premium-pricing or the high-CSR firm as the top firm on the legitimacy judgment, as well as on the behavioral item of the preferred choice of the most successful bidder for the project.

**Table 6:** Study 3 – Proportion of Participants Ranking the Premium-Pricing or High-CSR Firm as the Top One (Standard Deviations in Parentheses)

	Premium Pricing Signal			High CSR Engagement Signal		
	Market Logic Priming (*n* = 50)	Family Logic Priming (*n* = 49)	Overall (*n* = 99)	Market Logic Priming (*n* = 50)	Family Logic Priming (*n* = 49)	Overall (*n* = 99)
Sociopolitical legitimacy	0.69 (0.34)	0.39 (0.24)	0.55 (0.32)	0.91 (0.23)	0.80 (0.23)	0.86 (0.24)
Preferred choice	0.78 (0.42)	0.35 (0.48)	0.57 (0.50)	0.86 (0.35)	0.80 (0.41)	0.83 (0.38)

*Note*: Since some firms in our experiment had both a premium price and a high CSR engagement, the combined probabilities of ranking the premium-pricing or high-CSR firm as the top choice exceed 1.

First, Table 6 shows that the CSR signal across the two primes receives more positive attention from participants than the premium pricing signal, and this pattern is observed with respect to both sociopolitical legitimacy (0.91 vs. 0.69, *z* = 2.75, *p* < .01, 0.80 vs. 0.39, *z* = 4.13, *p* < .01, 0.86 vs. 0.55, *z* = 4.78, *p* < .01 for market logic, family logic and overall, respectively), and the preferred choice of the firm (0.86 vs. 0.78, *z* = 1.04, ns, 0.80 vs. 0.35, *z* = 4.51, *p* < .01, 0.83 vs. 0.57, *z* = 3.99, *p* < .01 for market logic, family logic, and overall, respectively). While we did not formulate any hypotheses with respect to relative strength of the two signals, it is conceivable that the pricing signal is probably more ambiguous than the CSR signal in general, as price premium may have multiple explanations—from superior quality and more socially responsible behavior to inefficiency and corporate greed. This ambiguity may have diluted the effect of the price premium on evaluations.

As in Study 2, we tested the proposed effect of pricing and CSR engagement on sociopolitical legitimacy evaluations. First, to test H1a, we checked whether the proportion of participants who ranked the moderate premium-pricing firm as the most legitimate firm is significantly higher than the random probability of 1/3. Overall, the proportion was 55%, and it was significantly higher than the random chance of 1/3 (*p* < .001), thus lending support to H1a and replicating the results of Study 2.

Similarly, to test H2, which predicts that CSR engagement is positively related to sociopolitical legitimacy evaluations, we examined whether the proportion of participants who ranked the high-level CSR engagement firm as the most legitimate firm is significantly higher than the random-chance probability of 1/2. Overall, the proportion was 86% and was significantly higher than the random chance of 1/2 (*p* < .001), thus lending support to H2.

Next, we tested the proposed moderation effect of the two institutional logics on the relationship between pricing strategy and legitimacy judgments, as outlined in H3. One-way ANOVA tests show that, compared to those participants who were in the family logic priming condition, the proportion of participants in the market logic priming who ranked the premium-pricing firm as the most legitimate firm, as well as the most favorable firm for the project, is significantly higher (0.69 vs. 0.39, *p* < .001, and 0.78 vs. 0.35, *p* < .001, respectively). Thus, these results largely replicate the moderation effect found in Study 2 and lend strong support to H3.

We performed the same set of tests for H4, which predicted that, relative to the market logic, the family logic will have a stronger moderating effect on the relationship between the CSR signal and legitimacy judgments. To our surprise, in contrast to the moderating results reported above for the pricing signal and the market logic (H3), one-way ANOVA tests show that among participants primed with the family logic, the proportion of participants who ranked the high-CSR firm as the top firm in terms of sociopolitical legitimacy was significantly lower than among participants primed with the market logic (0.80 vs. 0.91, *p* = .03). Lastly, there was no significant difference across the two priming treatments in terms of the proportion of participants who chose the high-CSR firm as the most favorable firm (0.86 vs. 0.80, ns). In other words, the effect of the CSR signal on legitimacy judgments and the final choice of the firm under the market priming was fairly high and in no way inferior to its effect under the family logic priming. This is in stark contrast with the effect of the pricing signal, which was much greater under the market logic priming than under the family logic priming (see the discussion of H3 above). These unexpected findings are summarized in Table 7 below.

**Table 7:** Study 3 – Hypothesized vs. Observed Moderating Effects of Institutional Logics Priming

Signal	Priming	Hypothesized Effect on Moderation	Observed Effect on Moderation
Moderate pricing premium	Market logic	Stronger (H3)	Supported (significant, *p* < .001)
Moderate pricing premium	Family logic	Weaker (H3)	Supported (significant, *p* < .001)
CSR engagement	Market logic	Weaker (H4)	Not supported as the effects of CSR signal are statistically the same (strong) under market and family logic priming.
CSR engagement	Family logic	Stronger (H4)	

It is interesting to note that the prime used to activate the market logic in the minds of participants did not contain any mentions of CSR or sustainability. However, the participants’ reaction to the CSR signal suggests that a cognitive schema of CSR engagement has been activated in their minds as a part of the market logic. At the same time, as expected, we have observed that a price premium was not much valued as a signal of underlying qualities of the firm by participants primed with the family logic.

Lastly, we explored whether sociopolitical legitimacy judgment mediates the effect of pricing/CSR engagement on the final choice of the bid winner. Similar to what was observed in Study 2, a proportion of the participants significantly higher than 1/3 (57% and 83%, *p* < .001 for both cases) chose the premium-pricing and high-CSR firm, respectively, as their most favorable firm for the project, which establishes the main effects for the mediation analysis. Using the same analytical framework as outlined in Study 2 for mediation analysis, random effects regression models and bootstrap analyses (Preacher et al., 2007) showed that the proposed mediator—sociopolitical legitimacy—significantly carries the influence of pricing to the most preferred choice of the firm. For the pricing signal (H5), these tests showed that legitimacy judgment mediated the relationship between the effect of pricing (effect = 0.15) on the choice of the firm. The effects were significant, as the 95% confidence interval ([.05,.25], bias-corrected) does not include zero, providing strong evidence of the presence of a mediating effect of sociopolitical legitimacy judgment on the relationship between moderate premium pricing and the choice of the firm, lending support for Hypothesis 5.

With respect to the CSR engagement signal (H6), the results were similar. Specifically, through sociopolitical legitimacy judgment, the indirect effect of CSR engagement (effect = 0.97) on the choice of the firm was significant. The 95% confidence interval for the mediator ([.83, 1.12], bias-corrected) does not include zero, thus lending support for Hypothesis 6. Our findings from Study 3 are summarized in Figure 6.

**Figure 5:**
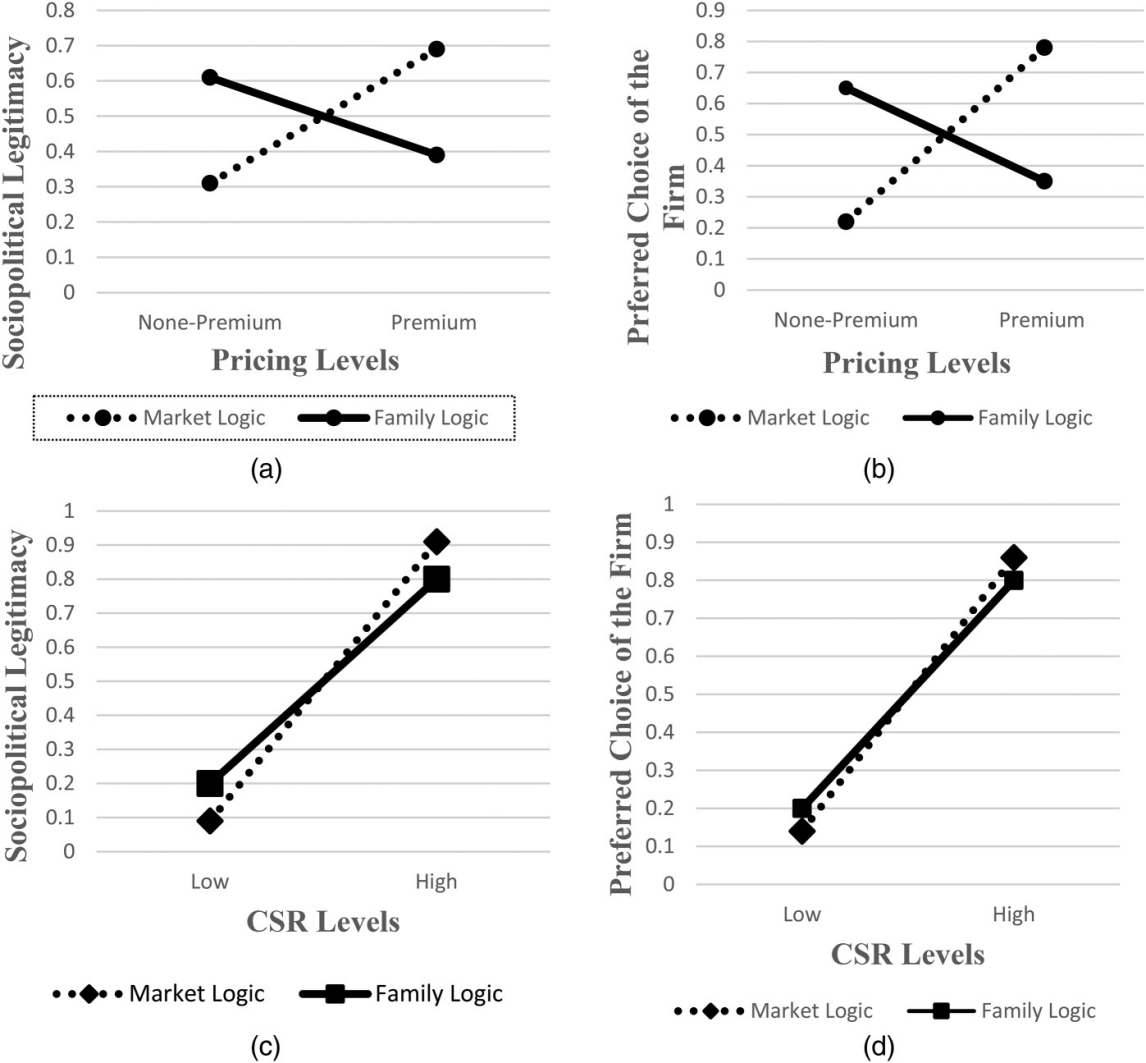
A, B, C and D: Study 3 – Proportion of Participants Ranking the Premium-Pricing Firm as the Top Choice

**Figure 6:**
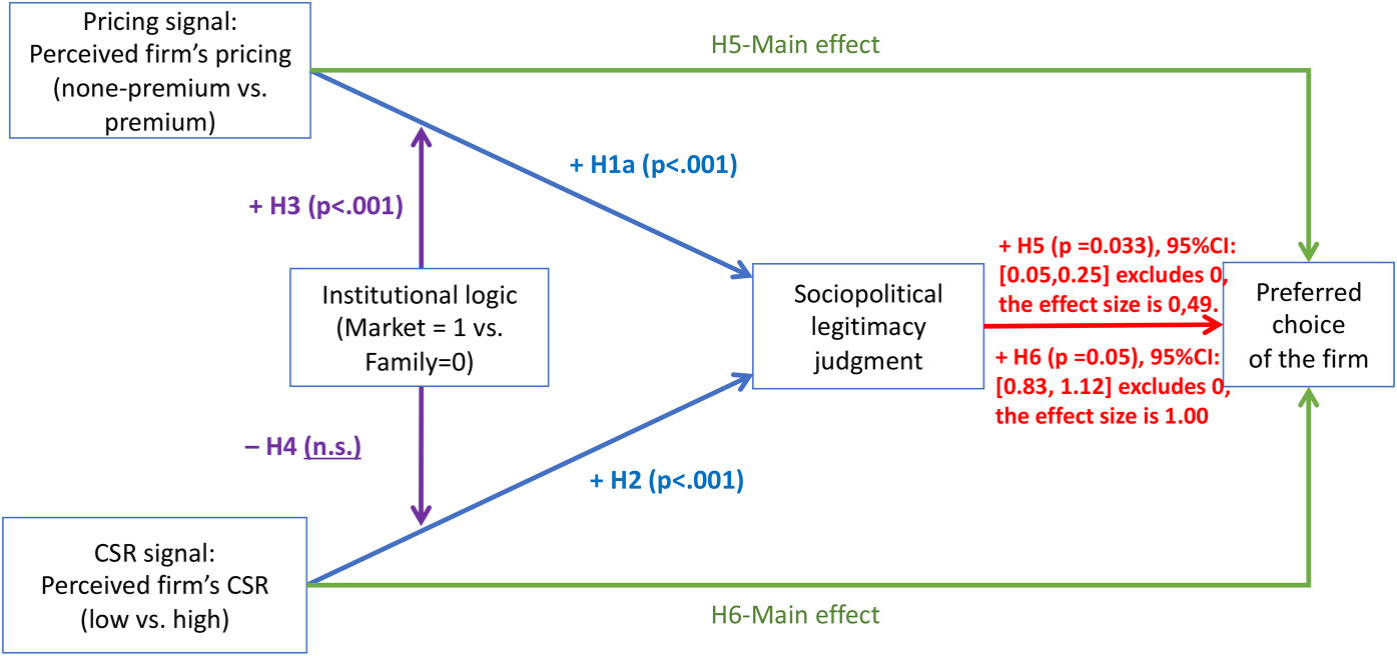
An Overview of Study 3 Findings

While we did not formally theorize or hypothesize whether and to what extent CSR engagement may have a synergistic effect on perceptions of premium-pricing, such an effect was plausible. In other words, it is conceivable that CSR engagement is seen as a validating and commendable justification for the premium pricing. To explore this conjecture, we conducted an *ex post* analysis for a possible interaction effect between the CSR engagement and the premium pricing signals. The results show that, under both the market logic and the family logic, perceived high CSR engagement indeed strengthened the positive relationship between premium pricing and legitimacy judgment (*p* < .01) as well as the positive relationship between premium pricing and the most favorable choice of the firm (*p* < .01). The presence of this effect further confirms that CSR engagement is valued by those primed with the market logic, as noted above.

## Discussion and Conclusion

The results presented in these studies jointly confirm that pricing serves as an important signal to evaluators and affects their judgments of the firm's sociopolitical legitimacy, as well as their choice of the firm. Study 1 confirmed the presence of an inverted U-shaped relationship between the price level and legitimacy judgments, where moderate pricing is associated with increased legitimacy, while extreme levels of pricing negatively affect legitimacy judgments. Similarly, firm's CSR engagement was found to be a significant signal that influenced both the evaluators’ assessment of the firm's sociopolitical legitimacy and their final choice of the firm for the project. These observations indicate that both CSR and moderate premium pricing signals have a direct positive effect on the behavioral intentions of the evaluators.

Studies 2 and 3 revealed a significant effect of institutional logics on sociopolitical legitimacy judgments, suggesting that, depending on which institutional logic is activated in the evaluator's mind, the evaluator may render different legitimacy judgments about the organization and even make different choices among the available alternatives. These results largely corroborate findings by Benard et al. (2019) that institutional logics priming can affect individuals’ judgments in an ambiguous judgmental task.

The unexpected, intriguing finding from our Study 3 was that CSR engagement was perceived as an important positive signal not only by those primed with the family logic but also by those primed with the market logic. Our findings on the effects of CSR signal under the market logic priming suggest that in the minds of our participants, the cognitive schema of CSR engagement is part of their understanding of the market logic, which makes CSR engagement work as an important signal of a firm's underlying qualities for those primed with the market logic as well. While counterintuitive and unexpected, these findings are in line with the observations by Ioannou and Serafeim (2015: 1054), who showed how the attitude toward CSR among financial analysts has undergone a fundamental change, and “over time and leading to 2007, analysts issue increasingly less pessimistic and, eventually, optimistic recommendations for firms with higher CSR scores.” In what Ioannou and Serafeim (2015) described, it was not that the market logic was replaced by some other institutional logic in financial markets, but the ideology component of the market logic changed from Friedman’s (1970) shareholder primacy to a more balanced, multiple-stakeholder perspective, which recognizes and values CSR.

These results also provide important insights for our understanding of the cognitive foundations of institutional logics. Studying how people primed with different logics react to the same stimuli allows us not only to demonstrate the power of institutional logics but also to explore how people understand a particular institutional logic, what cognitive and normative elements the logic includes for them. Glaser et al. (2017: 41) argued that individuals cognitively represent institutional logics as associated networks of logic components—entity and event schemas. Our Study 3 revealed that for the research participants involved in our study, the schema of CSR engagement forms an integral part of their understanding of the market logic: Although the market-logic prime did not contain any allusions to CSR, sustainability, or any other socially oriented concerns, the CSR engagement signal significantly and positively affected their perceptions of the firm. At the same time, participants primed with the family logic were much less sensitive to the market-based pricing signal.

Understanding the components of a particular institutional logic and how they differ from components of other logics is essential for advancement of research on interactions between institutional logics. Since institutional logics are not static and are theorized to constantly evolve, interact with other logics, and form new, hybrid logic types (Busco et al., 2017; Haveman & Rao, 2006; Ioannou & Serafeim, 2015; Jay, 2013; Thornton & Ocasio, 1999), in the absence of empirically grounded understanding of which socio-cognitive elements belong to which logic, researchers have no empirically verifiable way to identify which of the three situations described above they are facing. Experimental techniques similar to the one used in our Studies 2 and 3 hold great potential for investigating the components of institutional logics and addressing this serious shortcoming of the extant research on institutional logics.

Finally, our study suggests important implications for managerial practice and business education. As our study empirically showed that the cognitive schema of CSR engagement can form part of the market logic in the minds of the new generation of employees and managers, business education can play an important role in developing and strengthening this association. Furthermore, the institutional logics theory informed with our findings [cf. the principles of “analytical generalization” in Bitektine et al. (2018) and Yin (2010)] would suggest that business education can be more effective at advancing the goals of CSR and sustainable development if it focuses not on direct attempts to replace the market logic with some socially oriented logic (e.g., family, community, environmental, etc.) in business settings but rather on integration of the cognitive schema of CSR engagement into the market logic. Since institutional logics are activated in the presence of appropriate contextual cues (Benard et al., 2019; Glaser et al., 2017), the activation of a logic is predicated on the occurrence of such cues in the environment (Bargh, 2002). The business context abounds with contextual cues for activation of the market logic in the minds of employees and managers, while cues associated with different socially oriented logics are less present in this context and hence are less likely to be activated when corporate decisions are made. However, the presence of the association of the cognitive schema of CSR engagement with the market logic (as observed in our study participants) would allow the CSR engagement schema to be activated in business contexts as part of the market logic—that is, activated by the contextual cues that are highly prevalent in business settings. This suggests that the goals of CSR and sustainable development can be better achieved not by trying to invoke some socially oriented logic in business contexts but rather by redefining the understanding of what elements or schemas the market logic includes in such a way that the cognitive schema of CSR engagement becomes an integral component of the market logic for all actors operating in business settings.

### Limitations and Future Research

Several aspects of our experimental research deserve attention. First, since the logic of the laboratory experiment necessarily requires controlling for possible confounding factors, the resulting laboratory environment is designed to minimize possible confounding influences normally present in more complex real-world social settings. As a result, although the experimental set-up allows us to detect causal relationships, it may risk reduced external validity. Nevertheless, the methodology we employed created a controlled laboratory environment, enabling us to identify and isolate the effects of social signals, such as firms’ pricing and CSR engagement, and explore how the activation of a particular institutional logic (market or family) in evaluators’ minds affects their legitimacy judgments and behavioral intentions (Ajzen, 1991).

Second, we sampled from a population of undergraduate Commerce students. While such a sampling choice is often seen as limiting the generalizability of findings, we believe that in the context of this research sampling undergraduate business-school students was warranted, given our interest in how the new generation of employees and managers understands different institutional logics and how it reacts to specific social signals. Our study participants are already fully participating in our economy as consumers, full- or part-time employees, or are soon to enter the job market. Furthermore, as volunteers and representatives of interest groups or the general public, many of them have already acquired experience of engagement with social issues and participation in public policy decision-making, which matches the context of our study. Thus, the findings reported in this paper reflect the perspective of the new generation of employees and managers, and the generalization of our findings to other populations, such as those who are older and/or have more senior managerial responsibilities, should not be undertaken without further empirical research.

It is also conceivable that different types of institutional logics differ in their affinity with various subtypes of legitimacy. For example, Suchman (1995: 571) discerns pragmatic (“based on audience self-interest”), moral (“based on normative approval”), and cognitive (“taken-for-granted”) legitimacy subtypes. In our studies, we focused only on normative approval aspects of legitimacy (i.e., “moral” or “sociopolitical” legitimacy), while the affinity of the self-interest-based market logic with also self-interest-based pragmatic legitimacy was beyond the scope of our study. However, with the development of psychometrically valid measures that can differentiate moral and pragmatic legitimacy, the exploration of relationship of institutional logics with specific subtypes of organizational legitimacy can become a promising direction for future research.

Future research on the influence of institutional logics on judgments and actions should also explore the effects of other kinds of social signals and the mediating role of other types of institutional logics identified in the literature, such as professional logic (Freidson, 2001; Pahnke et al., 2015), bureaucracy logic (Jancsary et al., 2017), entrepreneurial logic (Miller et al., 2011), etc. It is also conceivable that other kinds of socially oriented logics, which share the emphasis on empathy, cooperation, the common good, and concern for others (e.g., community logic or environmental logic), would create a similar contrast with the market logic with respect to premium pricing and CSR signals. The validation of this conjecture also requires further empirical research.

Furthermore, experiments similar to the ones described in our Studies 2 and 3 have the potential to shed light on the actual content of institutional logics by revealing which signals resonate with individuals primed with a particular institutional logic. Our observations on the effects of CSR signal under the market logic priming and the effects of pricing signals under the family logic priming may thus open a new direction for empirical research on the content and composition of different institutional logics. The importance of this research agenda should not be underestimated: We already know that institutional logics constantly evolve (Besharov & Smith, 2014; Pache & Santos, 2013; Thornton et al., 2012), and our ability to empirically measure changes in their constitutive elements, such as implicit ideologies, cognitive schemas, goals, and means to achieve these goals, is essential for further advancement of research on institutional logics, their interactions, and their compatibility with each other.

## Appendix Table A1. Socially-Oriented Institutional Logics in the Literature

[Table table8-01492063211070274]

**Table table8-01492063211070274:**

Logic Type	Description (Emphasis Added)
Community logic	“…the community logic could be defined on the basis of *strong, affective, and enduring ties* among members of *small and bounded groups* … the community logic may be associated with *stronger community norms*, more *meaningful connections* and *richer social ties* with local individuals and organizations, and greater *reputational investment* of an organization's leaders, when they have more at stake than simply a financial investment.” (Almandoz, 2012: 1382) “The community logic bases identity on *emotional connections, ego-satisfaction and reputation* within a *common geographic boundary*, holds beliefs in *trust and reciprocity*, commits to *community values* and ideology, and develops strategies on status and honour of members.” (Hua, Chen, & Prashantham, 2016: 381) “Community is associated with a *sense of belonging* and *connection to something greater than one's self* <…>, *caring orientations* toward the community and its members <…>, and—ideally—the formation of relationships that are holistic, affective, and of value in themselves” <…>. The value society places on these *affective connections* is evident in community's status as a goal to be achieved <…>, as something worthy of preservation …” (Pirkey, 2015: 140) “…community logic is characterized by *group membership, personal investment in the group, relations of affect, loyalty, common values, and reciprocity* <…>” (Venkataraman et al., 2016: 710)
Family logic	“…associated with *notions of community* and *unconditional loyalty* to family members” (Friedland & Alford, 1991: 248) “In family firms, family actors are primary constituencies of one another. Their *close affective ties* may evoke in them familial attitudes and agendas even in a business context.” (Miller et al., 2011: 3) “…*nurturing*, generativity, and *loyalty* to the family” (Miller et al., 2011: 4) “*Personal attitude, dedication*, and the cultural fit between the CEO, the firm, and the family are valued higher than the individual's sole competence because the future leader of the firm is not only required to be ‘CEO’ in a corporate sense but, in particular, a ‘*family nurturer*’ (Miller et al., 2011) who secures the *well-being* of the numerous family members and future generations. Given *reciprocity norms* prevailing in the family <…>, the committed successor is likely to care about the various interests of family stakeholders and to preserve the *family's legacy*.” (Richards, Kammerlander, & Zellweger, 2019: 334) “Family logic focuses attention on successors’ *loyalty to the family and its assets*…” (Richards et al., 2019: 347) “Family influence alters the reference point for making decisions, which is related to a family's intention to *protect its endowment of socioemotional wealth*” (Dal Maso, Basco, Bassetti, & Lattanzi, 2020: 1551) “…individuals develop entity schemas for persons related to roles such as *father, mother, child, brother, sister, or friend*. Individuals develop entity schemas for objects associated with the family such as toys, minivans, a couch, or a dining room table. Individuals develop entity schemas for places such as a *home, a backyard, or a living room*. Additionally, individuals develop event schemas of scripts such as a *family dinner* or a *birthday party* and generalized representations of histories such as the ‘good-for-nothing’ relative or the ‘profligate father and suffering mother.’” (Glaser et al., 2017: 42)
Civic logic	“…emphasizes civic *solidarity* within the polity” (Lamont, 2012: 10) “…emphases on *rights, equality* and *the collective good*” (Anagnostopoulos, Lingard, & Sellar, 2016: 3) “…*values the collective over individual welfare* and *emphasizes law, rights, and issues of equity*. Tests of worth assess *equality and solidarity*.” (Anagnostopoulos et al., 2016: 6) “…civic arguments … emphasize collective responsibility” (Anagnostopoulos et al., 2016: 10)
Collective activity logic	“…meant to represent the *common interests of all its members*” (You & Guzman, 2019: 472) “The logic of collective activity, particularly the search for representational influence drives associations to seek as high a proportion as possible of members from their relevant sector or area of interest so as to maximise their legitimacy to *speak on behalf of the sector as a whole*: to be encompassing. <…> The logic of collective activity is to be contrasted from that of individual services because it is seeking to *address a problem of associability and collective action.*” (Bennett, 2000: 18)
Corporatist logic	“…the fundamental *logic of consensus building* to maintain an *organic rather than an atomistic community*. Key elements of the interinstitutional system (Thornton et al., 2012) support organizations in developing structures emphasizing *collective duties rather than individual rights and interests* within the national context. <…> a more corporatist logic supports a limited functional role of *the self as a member of a collective*, in contrast to a more entrepreneurial role supported by pluralism” (Vasudeva et al., 2015: 832) “Institutional infrastructures built around high corporateness carry an institutional logic in which the individual is expected to play a limited and *conformist role*. Individuals arc incorporated into the society and their organizations vis a vis their *specific functional roles in their respective collectives*.” (Luo, 2007: 284)
Social welfare logic	“…social welfare logic is structured around a predominant goal: making products and services available to *address local social needs*” (Pache & Santos, 2013: 979) “The social welfare logic further prescribes *democratic control* as the appropriate way to monitor strategy and operations. <…> *Social needs are localized*.” (Pache & Santos, 2013: 980) “The social welfare logic is *derived from the ideal type of the state* (Friedland & Alford, 1991; Thornton et al., 2012). It steers action toward *securing social and political order* (Dobbin & Dowd, 1997) at national or subnational levels (Marquis & Lounsbury, 2007), while considering a *plurality* of expressions <…>. In doing so, it can focus on overall employment and *wealth redistribution* issues (<…>; Thornton et al., 2012) or address more *local social needs* (Pache & Santos, 2013).” (Cristofini, 2021: 32) “…social welfare logic is based on the idea of *communitarianism*, *altruism*, and the *fulfillment of local social needs*…” (Risi, 2020: 1411) “…the social welfare logic, which has long been associated with a *nonprofit organizational form*, emphasizes *addressing social issues through democratic control*…” (Litrico & Besharov, 2019: 346) “Most frequent words in project descriptions, by logic type: Social Welfare Logic: “*social, community, grant, support, nonprofit, disabilities, assist, thrift, needs, society, help, public, education, educational, housing, arts, school, care*” (Litrico & Besharov, 2019: 347)
